# Rising Trends in Hospitalizations for Cardiovascular Events among Young Cannabis Users (18–39 Years) without Other Substance Abuse

**DOI:** 10.3390/medicina55080438

**Published:** 2019-08-05

**Authors:** Rupak Desai, Hee Kong Fong, Kaushal Shah, Vikram Preet Kaur, Sejal Savani, Kishorbhai Gangani, Nanush Damarlapally, Hemant Goyal

**Affiliations:** 1 Division of Cardiology, Atlanta Veterans Affairs Medical Center, Decatur, GA 30033, USA; 2 Division of Cardiovascular Medicine, University of California Davis Medical Center, Sacramento, CA 95817, USA; 3 Public Health, Western Kentucky University, Bowling Green, KY 42101, USA; 4 Smell & Taste Treatment and Research Foundation, Chicago, IL 60611, USA; 5 Public Health, New York University, New York, NY 10010, USA; 6 Department of Internal Medicine, Texas Health Arlington Memorial Hospital, Arlington, TX 76012, USA; 7 Department of Health Sciences, Coleman College of Health Sciences, Houston, TX 77030, USA; 8 Division of Gastroenterology and Hepatology, The Wright Center for Graduate Medical Education, Scranton, PA 18503, USA

**Keywords:** cannabis, marijuana, cardiovascular disease/events, young, trends, myocardial infarction, venous thromboembolism, arrhythmia, stroke, mortality

## Abstract

*Background and objectives:* Modern-day epidemiologic data on the risk and shifting landscape of occurrence of cardiovascular events in cannabis users remain inadequate and rather conflicting, especially amongst the young adult population. Furthermore, the problem of polysubstance use among youth is challenging for healthcare professionals and policy-makers. Previous studies report higher risk of concomitant use of tobacco, alcohol, cocaine, and amphetamine in young cannabis users. However, most of these studies did not eliminate the confounding effects of concomitant other substance abuse while assessing the incidence and outcome of cardiovascular events in cannabis users. *Materials and methods:* Using weighted discharge records from the National Inpatient Sample (NIS) from 2007–2014, we assessed the national trends in hospitalizations for major cardiovascular events including acute myocardial infarction (AMI), arrhythmia, stroke, and venous thromboembolic events (VTE) among young cannabis users (18–39 years), excluding cases with concomitant substance abuse with alcohol, tobacco, cocaine, and amphetamine. *Results:* Of 52.3 million hospitalizations without other substance abuse, 0.7 million (1.3%) young adults were current/former cannabis users. Among young adults without concomitant substance abuse, the frequency of admissions for AMI (0.23% vs. 0.14%), arrhythmia (4.02% vs. 2.84%), and stroke (0.33% vs. 0.26%) was higher in cannabis users as compared to non-users (*p* < 0.001). However, the frequency of admissions for VTE (0.53% vs. 0.84%) was lower among cannabis users as compared non-users. Between 2007 and 2014, we observed 50%, 79%, 300%, and 75% relative increases in hospitalizations for AMI, arrhythmias, stroke, and VTE, respectively, among young cannabis users as compared to non-users, showing relatively inferior or no ascent in the rates (*p*_trend_ < 0.001). *Conclusions:* The rising trends in hospitalizations for acute cardiovascular events among young cannabis users without concomitant other substance abuse call for future prospective well-designed studies to assess cannabis-related short-and long-term cardiovascular implications while simultaneously developing focused interventions towards raising awareness among the young population regarding the potential deleterious effects of cannabis use.

## 1. Introduction

Amidst legalization and decriminalization of recreational and medical use, cannabis (marijuana) has garnered numerous new users over the past few years and its usage is expected to rise even further by youth in the near future [1,2]. The patterns of polysubstance use among youth has remained a major concern for healthcare professionals and policy-makers over the last decade. Previous studies report heavy concurrent use of tobacco, alcohol, and cocaine beside cannabis by young adults [3,4,5]. With regard to the risk and prevalence of the cardiovascular diseases (CVD) in young cannabis users, modern-day epidemiological data remains scarce and is conflicting. Only a few studies have evaluated the association of cannabis use, CVD, and stroke in hospitalized patients [1,6,7,8,9]; even so, most of these studies did not examine the confounding effect of other substance abuse like tobacco, alcohol, or stimulant substances like cocaine or amphetamine, therefore, we proposed to assess the national trends in the hospitalizations for major cardiovascular events including acute myocardial infarction (AMI), arrhythmia, stroke, and venous thromboembolic events (VTE) among young cannabis users of age 18–39 years, while excluding hospitalizations with concurrent history of other substance abuse.

## 2. Material and Methods

The National Inpatient Sample (NIS) is the largest all-payer inpatient healthcare database in the United States (US), yielding 35 million inpatient records each year when weighted [10]. The NIS is developed by the Agency for Healthcare Research and Quality (AHRQ)-funded Healthcare Cost and Utilization Project. The current study was exempt from institutional review board approval due to the deidentified nature of the NIS datasets. The NIS databases (2007–2014) were analyzed to identify hospitalizations among adult cannabis users aged 18–39 years. We excluded cases with comorbid use of alcohol, tobacco, cocaine, and amphetamine. We assessed the trends in hospitalizations for AMI, arrhythmia, stroke, and VTE by using previously used the International Classification of Diseases, Ninth Revision, Clinical Modification (ICD-9-CM) codes as detailed in prior studies [1,6,7,11,12]. We also compared the baseline demographics, and in-hospital outcomes including all-cause mortality, the average length of stay (LOS) (days), and hospital charges between the cannabis users and non-users. Categorical and continuous data were assessed using Pearson’s Chi-square test and Student’s *t*-test, whereas trends were assessed by linear-by-linear association test using discharge weights in SPSS v22 (IBM Corp., Armonk, NY, USA). Statistical significance was measured at a two-sided *p*-value < 0.05.

## 3. Results

**Figure 1:**
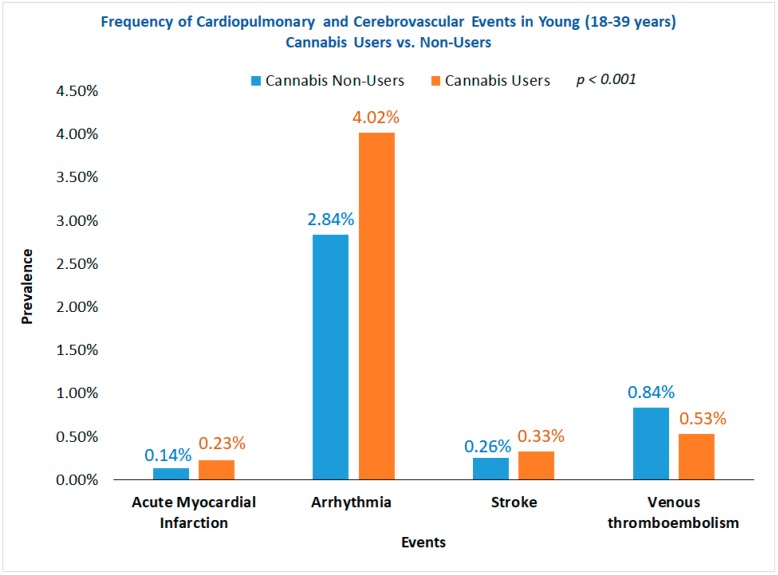
Frequency of cardiopulmonary and cerebrovascular events in young (18–39 years) cannabis users vs. non-users.

**Figure 2:**
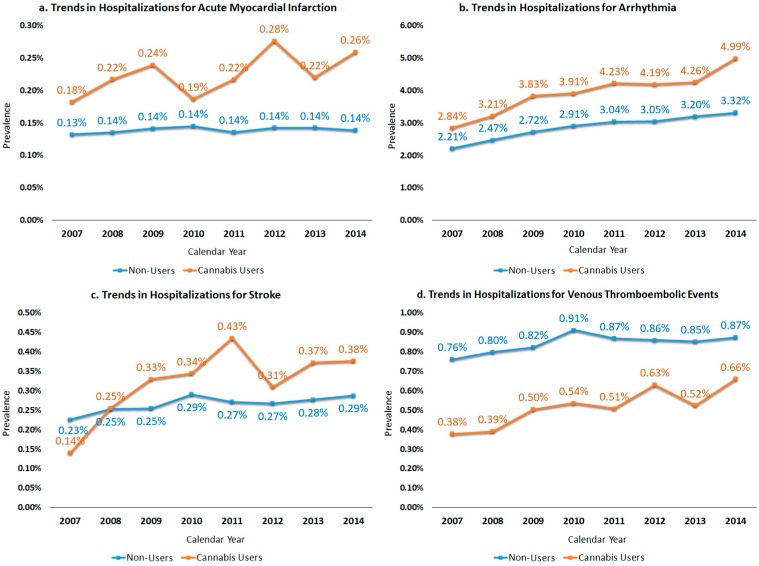
Trends in hospitalizations for major cardiovascular and cerebrovascular events among cannabis users vs. non-users of age 18–39 years, excluding cases with concomitant substance abuse (alcohol, tobacco, cocaine, and amphetamine). (**a**) Trends in hospitalizations for acute myocardial infarction. (**b**) Trends in hospitalizations for arrhythmia. (**c**) Trends in hospitalizations for stroke. (**d**) Trends in hospitalizations for venous thromboembolic events.

We identified a total of 52,290,927 US nationwide hospitalizations among young patients (18–39 years) after excluding cases with concurrent history of use of alcohol, tobacco, cocaine, and amphetamine. Of these, 1.3% (*n* = 669,407) patients were former or current cannabis users. Cannabis users were relatively younger (mean age 26 vs. 28 years) male (48.8% vs. 16.3%, *p* < 0.001) patients in comparison to non-users (Table 1). The frequency of AMI (0.23% vs. 0.14%), arrhythmia (4.02% vs. 2.84%), and stroke (0.33% vs. 0.26%) were higher in cannabis users as compared to non-users (*p* < 0.001) (Figure 1). Surprisingly, the frequency of VTE (0.53% vs. 0.84%) was lower among cannabis users, but the trends in hospitalizations for VTE showed a greater relative increase among cannabis users (0.4% to 0.7%; 75% relative increase, *p*_trend_ < 0.001) as compared to non-users (0.8% to 0.9%; 12.5% relative increase, *p*_trend_ < 0.001) from 2007 to 2014. Trends in the hospitalizations for AMI, arrhythmia, stroke, and VTE are depicted in Figure 2. As compared to 2007, we observed 50%, 79%, 300%, and 75% relative increases in hospitalizations for AMI (0.2% to 0.3%), arrhythmias (2.8% to 5.0%), stroke (0.1% to 0.4%), and VTE (0.4% to 0.7%), respectively, in 2014 among young cannabis users as compared to non-users, showing relatively inferior or no ascent in the rates (*p*_trend_ < 0.001). Nevertheless, the all-cause inpatient mortality was lower (0.2% vs. 0.3%, *p* < 0.001) among young cannabis users compared to non-users. The average LOS was higher (mean 4.9 days vs. 3.4 days, *p* < 0.001) in cannabis users as compared to non-users, however, hospitalization charges were lower ($20,883 vs. $21,843, *p* < 0.001) compared to non-users. 

## 4. Discussion

With increasing reports suggesting concerning patterns of polysubstance abuse among youth, it is imperative to assess the influence of only cannabis use on cardiovascular health with the exclusion of confounding effects by concurrent substance abuse with tobacco, alcohol, cocaine, and amphetamine. To our knowledge, no studies have revealed trends in the cardiovascular events in the young lone cannabis users. 

In our study, the prevalence of AMI was higher (0.2% vs. 0.1%) among cannabis users as compared to non-users. The trends in AMI-related hospitalizations rose from 0.2% to 0.3% from 2007 to 2014 among cannabis users compared to the overall stable (0.1%) rate of admissions for AMI among patients even after excluding major confounders such as tobacco, alcohol, and other stimulant drug abuse (cocaine and amphetamine). There are isolated reported cases of AMI after heavy cannabis use [13,14], however, this is the first nationwide study reporting trends in AMI among young cannabis users. The literature proposes the possible detrimental effects of cannabis abuse on heart rate, blood pressure, cardiac output, left ventricular ejection time, and venous carboxyhemoglobin levels [1]. 

The most striking finding was the considerably higher prevalence (4.0% vs. 2.8%) and rising trends in the admission for arrhythmias among young cannabis users (from 2.8% to 5.0%) compared to non-users (from 2.2% to 3.3%) from 2007–2014. These findings are consistent with previous published studies showing that a considerable proportion of cannabis users experience in-hospital arrhythmias that are both fatal and non-fatal [6]. Moreover, time-based trends in the arrhythmia-related hospitalizations have never been reported in the past among young cannabis users.

Tobacco, cocaine, and alcohol are well-established risk factors for stroke in young adults [8,15]. However, stroke in young cannabis users have not been widely appraised in contemporary literature. The frequency of stroke-related admissions was marginally higher (0.33% vs. 0.26%) among cannabis users even after excluding cases with other substance abuse. Consistently, the admissions for stroke in young cannabis users (from 0.1% to 0.4%) surpassed non-users (from 0.2% to 0.3%) from 2007 to 2014. Rumalla et al. found that marijuana use was associated with a higher risk of stroke events [9]. However, a Swedish study did not find an increased risk of young onset of stroke with cannabis use [8]. 

Another interesting finding of this study is that despite an overall lower prevalence of VTE among cannabis users (0.5% vs. 0.8%) compared to non-users, cannabis users experienced expeditiously rising trends in hospitalizations (from 0.4% to 0.7%) for VTE compared to non-users (from 0.8% to 0.9%) from 2007 to 2014. Literature remains limited to isolated case reports reporting marijuana-associated pulmonary embolism [16,17].

This study also bears a few limitations that should be considered while inferring study findings. The mode and dose of cannabis use, smoke to event interval, and follow-up data were unknown. Prior reports have suggested that most (>85%) therapeutic users tend to use cannabis recreationally, therefore, we assumed that the present study was mainly inclusive of recreational users between 2007 and 2014 [18]. Despite earlier studies having successfully used ICD-9-CM codes to identify cannabis users, there are data showing overall low predictive values of administrative coding to assess the substance abuse along with a possibility of self-reporting bias to an extent [19]. Although this study could not establish direct causation between cannabis use and cardiovascular events in young adults, findings indicate a positive association considering rising trends in cardiovascular events even without any influence of concurrent substance abuse. Lack of patient-level clinical information of this study population did not allow for analyses to be adjusted for potential confounders, which should be taken into consideration while drawing any conclusions from this study. This is the largest study to date providing crucial national estimates on trends in CVD among young cannabis users, which should serve as a premise for future prospective randomized or case-control studies.

## 5. Conclusions

Concisely, this study uncovered growing rates and rising trends in hospitalizations for AMI, arrhythmia, and stroke from 2007–2014 among young cannabis users compared to non-users even without concurrent other substance abuse. Rates of VTE was lower among cannabis users as compared to non-users, with rising trends in VTE admissions in both cannabis users and non-users. In addition to control of polysubstance use, targeted interventions towards raising awareness of the potential harmful effects of cannabis among youth is crucial.

## Figures and Tables

**Table 1:** Baseline characteristics and in-hospital outcomes in young (18–39 years) cannabis users vs. non-users without concurrent substance abuse with tobacco, alcohol, cocaine, or amphetamine.

	Non-Users (*n* = 51,621,520)	Cannabis Users (*n* = 669,407)	*p*
Age (years) at admission, Mean ± SD	28.4 ± 5.9	26.2 ± 5.8	<0.001
Sex			<0.001
Male	16.3%	48.8%	
Female	83.7%	51.2%	
Race			<0.001
White	52.9%	48.4%	
Black	17.5%	34.6%	
Hispanic	20.0%	11.4%	
Asian or Pacific Islander	4.1%	1.0%	
Native American	0.8%	1.0%	
Others	4.7%	3.6%	
Comorbidities			
Congestive heart failure	0.5%	0.4%	<0.001
Chronic pulmonary disease	5.7%	10.3%	<0.001
Depression	4.4%	8.2%	<0.001
Hypertension	7.2%	8.9%	<0.001
Diabetes, uncomplicated	3.1%	2.6%	<0.001
Diabetes with chronic complications	0.8%	0.9%	<0.001
Dyslipidemia	2.1%	2.3%	<0.001
Obesity	6.6%	6.5%	0.002
Pulmonary circulation disorders	0.4%	0.3%	<0.001
Peripheral vascular disorders	0.2%	0.2%	<0.001
Renal failure	1.5%	1.2%	<0.001
Length of stay (days), Mean ± SD	3.4 ± 5.1	4.9 ± 7.5	<0.001
Total hospital charges, Mean	$21,843	$20,883	<0.001
All-cause in-hospital mortality	0.3%	0.2%	<0.001
Disposition of patient			<0.001
Routine	92.9%	88.3%	
Transfers to short-term hospital	0.9%	1.3%	
Other transfers (SNF, ICF, etc.)	1.7%	4.3%	
Home healthcare	3.2%	2.0%	
Against medical advice	1.0%	3.8%	

*p* < 0.05 indicates statistical significance. SNF—skilled nursing facility, ICF—intermediate care facility.
